# Correlation Study on Antibiotic Resistance and Antibacterial Activity of Soil Microorganisms in Lop Nur

**DOI:** 10.3390/microorganisms13092076

**Published:** 2025-09-06

**Authors:** Feng Wen, Qiannan Chen, Yingying Zhao, Xiaoting Zhang, Guo Yang, Hui Jiang, Zhanfeng Xia

**Affiliations:** Key Laboratory of Protection and Utilization of Biological Resources in Tarim Basin, College of Life Sciences and Technology, Xinjiang Production & Construction Corps, Tarim University, Alar 843300, China; fengwen0306@163.com (F.W.); 15128152130@163.com (Q.C.); 17699286136@163.com (Y.Z.); 19593170413@163.com (X.Z.); yguo1297@163.com (G.Y.)

**Keywords:** Lop Nur, extremophiles, multidrug resistance, resistant strains with antibacterial activity

## Abstract

Lop Nur, an extremely arid environment, harbors unique microbial resources and represents a potential reservoir for novel bioactive substances. With antibiotic resistance becoming an increasing global concern, the resistance traits of microorganisms in extreme habitats and their potential association with antibacterial activity remain poorly understood. This study aimed to investigate the diversity of soil microorganisms in Lop Nur, their resistance to norfloxacin, kanamycin, and amoxicillin, and their inhibitory activity against common pathogenic bacteria, thereby providing a scientific basis for the discovery of new antibacterial candidates. Surface soils from three sampling points in Lop Nur were inoculated onto Gao’s No.1 and LB media supplemented with different antibiotic regimens (single, pairwise, and triple combinations). Isolates were identified by 16S rRNA gene sequencing, their antibiotic resistance was assessed using the disk diffusion method, and antibacterial activity was evaluated using the agar well diffusion method. A total of 120 microorganisms were isolated, belonging to six phyla and nine genera, including 10 potential new species. The control group yielded the highest diversity (35 strains), whereas only 4 strains were recovered under triple-antibiotic treatment, demonstrating the strong selective effect of antibiotic stress. Resistance profiling showed that 88.14% of strains were resistant to amoxicillin, 64.71% to norfloxacin, and 60.68% to kanamycin, with multidrug resistance being widespread. Eleven strains exhibited antibacterial activity against five pathogens, including Staphylococcus aureus (maximum inhibition zone 53.51 mm), and nine of these strains also displayed antibiotic resistance, suggesting a potential association between resistance and antibacterial activity. Microorganisms isolated from Lop Nur displayed extensive resistance and notable antibacterial activity. Antibiotic stress strongly influenced the cultivable microbial isolates, facilitating the recovery of resistant strains with antibacterial potential. These findings provide a valuable reference for exploring microbial resources in extreme environments and highlight the potential link between antibiotic resistance and antibacterial activity.

## 1. Introduction

Lop Nur, located in the extremely arid northwest of China, has undergone drastic ecological changes from an inland lake to a desert, forming a unique “arid–saline–strong radiation” compound stress environment [1]. The annual average precipitation in this area is less than 20 mm, the soil moisture content is consistently below 5%, the pH value is as high as 8.5–9.5, the soluble salt content reaches 10–20 g/kg, and the ultraviolet radiation intensity is 2–3 times higher than the average level in other regions of China. This extreme habitat has given rise to microbial communities with special metabolic mechanisms, making it a potential treasure trove for the discovery of novel bioactive substances [2].

In recent years, microbial research in Lop Nur has gradually become a hot topic in extremophile biology. Wu Hongqi et al. (2009) first isolated 168 halophilic bacteria from Lop Nur salt lake, finding that Halomonas species accounted for 37%, and detected the activities of enzymes such as amylase and esterase [3], revealing the unique potential of microorganisms in this area for carbon and nitrogen metabolism. However, none of these studies have involved the analysis of antibiotic resistance. Globally, the problem of antibiotic abuse is becoming increasingly severe, and the resulting resistance crisis has gradually spread to natural ecosystems. Soil is a huge “resistance gene pool”; the dynamic balance between its inherent resistance (such as ancient resistance genes from 30,000-year-old permafrost bacteria) and acquired resistance is becoming the key to understanding the natural evolution of resistance [4,5,6,7]. In the Lop Nur habitat, which is minimally disturbed by humans but faces natural stresses such as high salinity and drought, whether microbial resistance is enriched through a “co-selection” mechanism (such as the co-expression of salt stress and antibiotic resistance genes) remains unreported.

Norfloxacin, kanamycin, and amoxicillin are commonly used antibiotics in clinical and agricultural fields [8,9], targeting DNA gyrase, ribosomes, and cell wall synthesis pathways, respectively [10]. In extreme environments, similar metabolic stresses may drive the co-evolution of microbial resistance genes and secondary metabolic genes through co-selection. However, there is still a lack of in-depth understanding of the distribution of multi-antibiotic resistance in microorganisms under the combined stress of drought and salinity, the mechanisms of multi-resistance, and their association with antibacterial activity.

Based on this, this study collected surface soil samples from three sampling points in Lop Nur, and used Gao’s No. 1 and LB media with different antibiotic combinations (single use, two-way combination and three-way combination) for isolation and culture. The microbial groups were identified by 16S rRNA gene sequencing. Drug resistance was detected by the disk diffusion method and the broth dilution method, and the antibacterial activity against five pathogenic bacteria was evaluated by the well diffusion method. The aim is to answer the following scientific question: Can different combinations of norfloxacin, kanamycin and amoxicillin screen and enrich specific drug-resistant groups through the “multiple pathway blocking” effect targeting DNA replication, protein synthesis and cell wall construction, and does this enrichment effect increase with the complexity of the antibiotic combination?

The research results will provide a scientific basis for the development of microbial resources in extreme environments and the analysis of the drug resistance–antibacterial activity relationship, while also offering a new perspective for exploring the functional adaptive evolution of microorganisms in extreme habitats.

## 2. Materials and Methods

### 2.1. Test Samples

**Figure 1:**
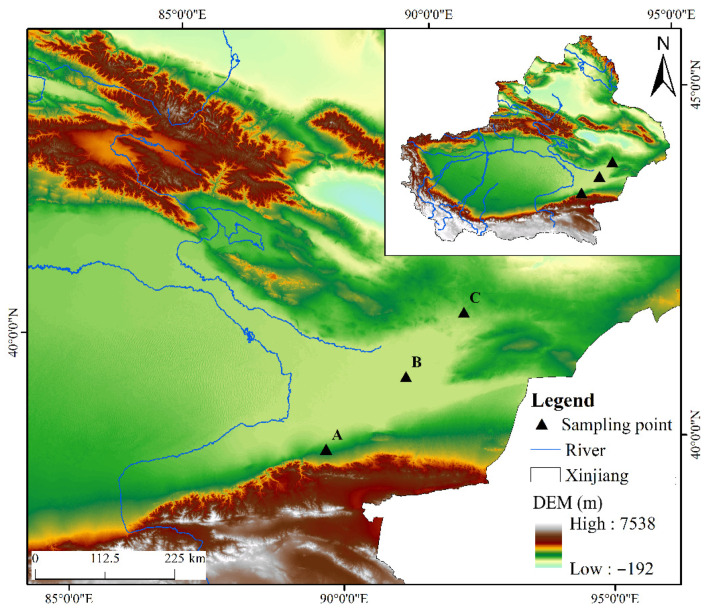
Sampling map of Lop Nur.

In July 2022, three representative sampling points were selected in the Lop Nur area and divided into three directions: north, south and center. At each sampling point, a five-point sampling method was adopted to collect soil samples ranging from 0 to 20 cm from the surface layer; the specific sample information is shown in Table 1 and Figure 1. After thorough mixing, the samples were placed in sterile sealed bags and stored at low temperatures before being brought back to the laboratory [11].

### 2.2. Extraction of Total DNA from Soil Samples and Analysis of Bacterial Community Structure Based on 16S rRNA Gene

Total bacterial DNA was extracted using a kit (OMEGA M5635-2, Michigan City, IN, USA), the DNA was quantified using Nanodrop, and the quality was checked by 1.2% agarose gel electrophoresis. The V3–V4 variable region of bacterial 16S rRNA was amplified using specific primers 799 F (5′-ACTCCTACGGGAGGCAGCA-3′) and 1193 R (5′-GGACTACHVGGGTWTCTAAT-3′). The PCR amplification was carried out in a 25 μL reaction system with the following conditions: 98 °C pre-denaturation for 2 min; 98 °C denaturation for 15 s, 55 °C annealing for 30 s, 72 °C extension for 30 s, for 25 cycles; and a final extension at 72 °C for 5 min.

The PCR amplification products were sent to Shanghai Personal Biotechnology Co., Ltd., for sequencing (http://www.personalbio.cn, accessed on 1 January 2025). High-throughput sequencing was performed using the Illumina Novaseq and Miseq platforms (Illumina, San Diego, CA, USA). The raw sequence data obtained were analyzed as follows: The samples were quality-checked, and the libraries were constructed. The Illumina Novaseq sequencing platform was used for analysis. The samples were split based on the Barcode sequence, and DADA2 was used for primer removal, filtering, denoising, assembly, and chimera removal to obtain high-quality sequences. Then, the high-quality sequences were classified into corresponding ASVs (amplicon sequence variants) using a feature classifier based on the sklearn naive Bayes algorithm and compared with the Silva database (Release132 http://www.arb-silva.de, accessed on 22 April 2025) [12,13] to obtain species classification information.

### 2.3. Isolation and Culture Methods

The collected soil samples were free of stones, plant residues, and other impurities, passed through a 2 mm sieve, and ground in a sterile mortar. Approximately 0.01 g of soil was taken and imprinted onto Gao’s No. 1 medium and LB medium supplemented with different antibiotic treatments using a sterile velvet cloth. The concentrations of the antibiotics were as follows: norfloxacin (0.0015 μg/mL), kanamycin (0.0625 μg/mL), and amoxicillin (0.004 μg/mL). These concentrations were selected based on our previous study on antibiotic effects in special habitats in Xinjiang [14] where they yielded optimal isolation efficiency, and were further confirmed by preliminary experiments in this work. Antibiotics were applied either singly, in pairs (norfloxacin + kanamycin, norfloxacin + amoxicillin, kanamycin + amoxicillin), or in a combination of all three, while maintaining the same concentrations. Three parallel plates were prepared for each treatment group, inverted, and incubated at 30 °C in a constant-temperature incubator for 24–48 h. Colony morphology was observed and recorded.

### 2.4. Antibiotic Resistance Detection Methods

The resistance of isolated microorganisms to norfloxacin, kanamycin, and amoxicillin was detected using the disk diffusion method (K-B method) and the broth dilution method. Single colonies were isolated, purified, inoculated into liquid medium and incubated at 37 °C with shaking (180 rpm). The growth was monitored spectrophotometrically, and the logarithmic phase was determined when the optical density at 600 nm (OD_600_) reached 0.6–0.8, typically after 6–8 h of incubation. The bacterial suspension was adjusted to a turbidity of 0.5 McFarland units (approximately 1 × 10^8^ CFU/mL). The suspension was spread evenly on the surface of MH agar plates using a sterile cotton swab. Antibiotic susceptibility disks containing norfloxacin (5 μg/disk), kanamycin (30 μg/disk), and amoxicillin (10 μg/disk) were placed on the plates. The plates were incubated at 37 °C for 18–24 h, and the diameters of the inhibition zones were measured. Resistance was determined according to CLSI standards [15]. Meanwhile, the minimum inhibitory concentration (MIC) was determined by the broth dilution method. Antibiotics were serially diluted and added to 96-well plates containing the bacterial suspension. After incubation at 37 °C for 24 h, the lowest drug concentration at which no bacterial growth was observed by the naked eye was defined as the MIC.

### 2.5. Method for Detecting Antibacterial Activity

*Staphylococcus aureus* ATCC 25923, *Escherichia coli* ATCC 25922, *Candida albicans* ATCC 10231, *Erwinia amylovora* ATCC 15580, *Enterococcus faecalis* ATCC 29212, and *Enterococcus faecium* ATCC 35667 were selected as indicator pathogens. The antibacterial activity of the fermentation broth of isolated microorganisms was detected by the well diffusion method. The well diffusion method was carried out as follows: The indicator pathogens were inoculated onto solid medium and spread evenly. A sterilized punch was used to make holes in the plates, and 100 μL of the culture supernatant was obtained by centrifugation at 10,000× *g* for 10 min at 4 °C to remove cells and debris, and then added into the wells. After incubation at 37 °C for 24 h, the diameters of the inhibition zones were measured.

### 2.6. Data Processing of Microbial Resistance Scores and Antibacterial Activity and Heatmap Generation

To systematically quantify the resistance level of each strain to antibiotics and its antibacterial ability against pathogenic bacteria, a resistance scoring system was established in this study, combined with visualization analysis methods for data processing and graphical presentation. The antibiotic susceptibility test results of each strain to various antibiotics were represented by three categorical variables, S (sensitive), I (intermediate), and R (resistant), and were assigned numerical codes, S = 0, I = 1, and R = 2, to reflect the increasing trend of resistance. The average resistance score of each strain was calculated, ranging from 0 to 2, with higher scores indicating stronger resistance. Subsequently, the mean numerical code of each strain across all antibiotics was calculated to generate a comprehensive resistance score, which was used as a continuous variable representing the strain’s multidrug resistance level.

To evaluate the antibacterial ability, the diameters of the inhibition zones (in mm) of each strain against all indicator pathogens were counted, and the mean inhibition zone diameter was calculated to reflect the strength of the broad-spectrum antibacterial effect. All raw data cleaning, variable standardization, merging, and format conversion were performed in R software (version 4.3.2) [16] using the tidyverse package. To visualize the resistance spectrum and antibacterial spectrum of the strains, heatmaps for resistance and antibacterial activity were drawn. The resistance heatmap had strains as rows and antibiotics as columns, with color gradients from light to dark representing sensitive, intermediate, and resistant states. The antibacterial activity heatmap had strains as rows and pathogenic bacteria as columns, with colors ranging from white (0 mm) to dark blue (maximum inhibition zone) indicating the strength of the antibacterial effect. Both used Euclidean distance and complete linkage for bidirectional clustering of rows and columns. To explore whether the drug resistance level of the strains is related to their inhibitory ability against pathogenic bacteria, this study constructed two quantitative indicators for each strain: resistance score and mean inhibition zone diameter. The former was obtained by converting the sensitivity categories (S: sensitive; I: intermediate; R: resistant) of the strain to multiple antibiotics into numerical values of 0, 1, and 2, and then taking the average of the scores for all antibiotics. The latter was the average of the inhibition zone diameters (unit: mm) of the strain against all indicator pathogenic bacteria, reflecting its overall antibacterial activity level. Spearman’s rank correlation analysis was used to evaluate the statistical correlation between the two, and a trend line was added to the scatter plot through linear regression to assist in visualization. The graphs were drawn using R language (version 4.3.2) and its graphic extension packages such as ggplot2 [17] and ggpubr [18].

## 3. Results

### 3.1. Bacterial Community Structure and Diversity Analysis Based on 16S rRNA Gene

#### 3.1.1. Bacterial Community Structure

**Figure 2:**
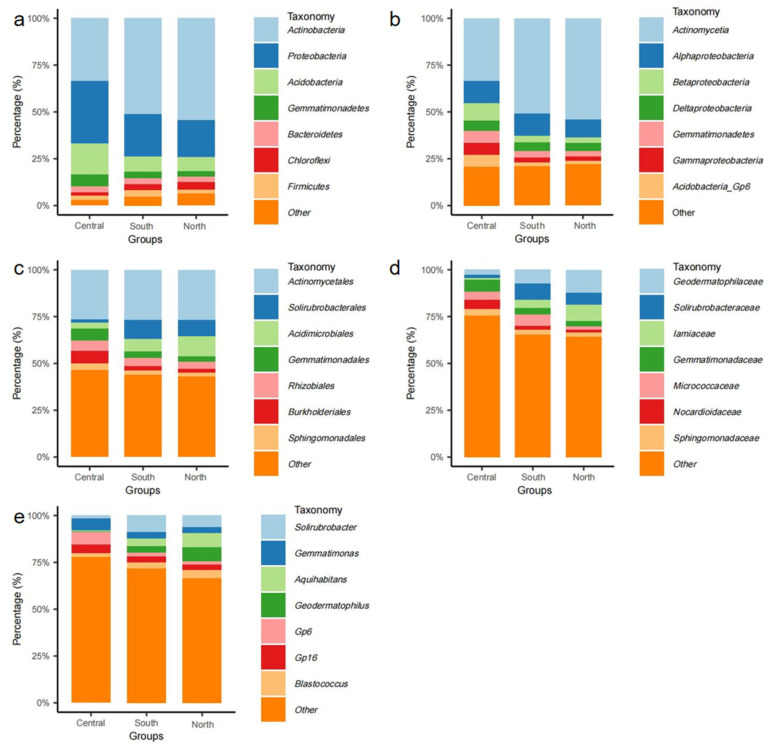
Taxonomic composition of bacterial communities in soil samples from different regions of the Lop Nur Basin. (**a**) Phylum level; (**b**) class level; (**c**) order level; (**d**) family level; (**e**) genus level.

To explore the compositional differences in soil microbial communities in different geographical locations of Lop Nur, microbial classification bar charts were drawn at the phylum, class, order, family, and genus levels (Figure 2). The results showed that there were obvious abundance variations among different locations for various groups:

At the phylum level (Figure 2a), *Actinobacteria* and *Proteobacteria* were the most dominant groups in the three sample sets. *Actinobacteria* had a slightly higher proportion in the central and north samples, while *Proteobacteria* was relatively more abundant in the south region. Additionally, *Acidobacteria* and *Gemmatimonadetes* were also distributed in all groups, but their proportions were slightly higher in the southern samples.

At the class and order levels (Figure 2b,c), *Actinomycetia* and *Solirubrobacterales* were the main classification units in all samples. *Actinomycetia* was most dominant in the central samples, while *Solirubrobacterales* had a higher abundance in the south region. *Gemmatimonadales* and *Acidimicrobiales* also had relatively higher proportions in the south and north samples, suggesting that these two groups may have strong adaptability to local environments.

At the family and genus levels (Figure 2d,e), *Geodermatophilaceae* and *Micrococcaceae* were significantly enriched in the central samples, while the south samples had a higher proportion of *Solirubrobacteraceae* and unclassified genera such as Gp6 and Gp16, indicating differences in microbial composition between the central and southern samples. Notably, *Aquihabitans* and *Blastococcus* had slightly higher abundances in the north samples, possibly reflecting the unique ecological niche adaptation characteristics of this region.

Overall, although the major phyla were distributed in all three groups, more significant distribution differences were observed at the lower classification levels (order, family, genus), suggesting that microbial communities in different geographical locations may have differentiated environmental adaptability and ecological functions.

#### 3.1.2. ASV Counts and Alpha Diversity Indices

High-throughput sequencing of soil samples yielded a total of 741,798 raw reads, from which 31,010 initial sequences and 397,229 high-quality effective sequences were obtained. Operational taxonomic units (ASVs) were clustered at a 97% similarity threshold, resulting in 4017 amplicon sequence variants (ASVs) using QIIME2 (version 2023.5).

**Figure 3:**
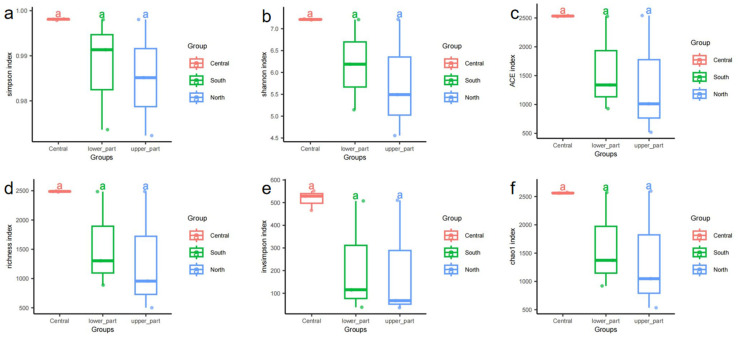
Comparison of alpha diversity indices under different culture conditions. (**a**) Simpson index; (**b**) Shannon index; (**c**) ACE index; (**d**) richness index; (**e**) Invsimpson index; (**f**) chao1 index. Different lowercase letters above the boxes indicate significant differences among groups at *p* < 0.05, while the same letters indicate no significant difference.

Although no statistically significant differences in alpha diversity indices (Simpson, Shannon, ACE, Chao1, Richness, and Invsimpson) were observed among the three sampling groups (ANOVA, *p* > 0.05; see Figure 3), distributional patterns revealed distinct ecological trends. The central group exhibited consistently higher and more centralized alpha diversity values with minimal variation, suggesting a more stable and uniform microbial community structure. In contrast, both the south and north groups showed greater dispersion in diversity values, particularly in the Shannon and Inverse Simpson indices within the south group, indicating a potentially more heterogeneous or complex microbial composition.

Additionally, higher Chao1 and ACE indices in the south and north groups suggest a greater presence of low-abundance or rare taxa, implying the existence of underrepresented microbial diversity in these regions. While statistical significance was not reached, these ecological patterns imply that the south and north regions may harbor higher microbial community heterogeneity and dynamic variability. In contrast, the central region, characterized by high diversity yet low dispersion, may represent a more mature and ecologically stable microbial assemblage.

### 3.2. Results of Microbial Isolation and Culture

#### 3.2.1. The Influence of Antibiotics on the Selective Isolation and Culture of Microorganisms

The blank control group isolated the largest variety and quantity of microorganisms, indicating that antibiotics have an inhibitory effect on the growth of microorganisms [19]. When antibiotics were used alone, the bacteria isolated from norfloxacin medium were mainly Gram-negative bacteria, while both Gram-positive and Gram-negative bacteria were isolated from kanamycin medium. Relatively few types of microorganisms were isolated from amoxicillin medium. When used in pairs or in combination with three antibiotics, the types and quantities of isolated microorganisms significantly decreased, and the effects of different combinations on the microbial community structure were different (Table 2).

#### 3.2.2. Strain Information

**Figure 4:**
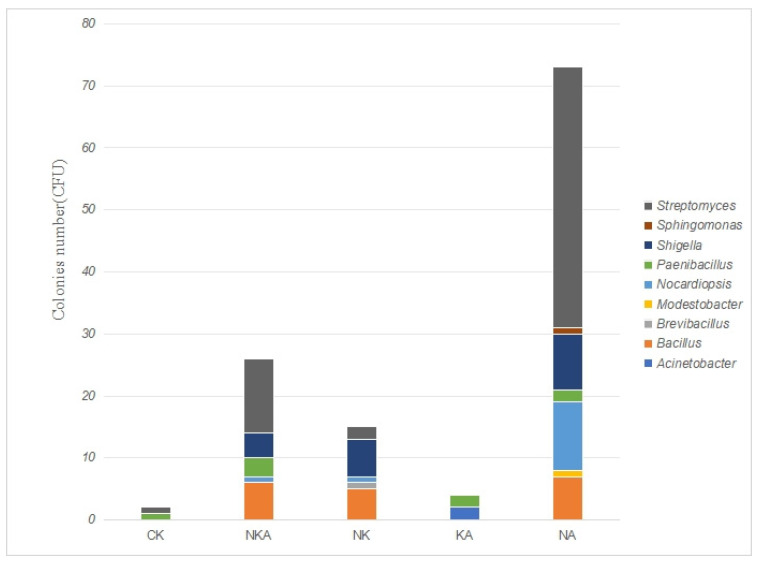
Genus-level distribution of microbial isolates obtained under different antibiotic treatments. NKA: norfloxacin, kanamycin and amoxicillin; CK: no antibiotics added; NK: norfloxacin and kanamycin; KA: kanamycin and amoxicillin; NA: norfloxacin and amoxicillin.

A total of 120 bacterial strains were isolated and identified from the soil samples of Lop Nur on media with different antibiotic combinations. Through 16S rRNA gene sequencing and sequence alignment analysis, they belonged to six phyla, four classes, seven orders, eight families and nine genera. The strain information is shown in Figure 4. The dominant phyla are *Actinobacteria* and *Pseudomonadota*. The dominant bacterial classes are *Actinomycetia* and Bacilli. The dominant bacterial orders are *Streptomycetales* and *Bacillales*. The dominant bacterial families are *Streptomycetaceae* and *Enterobacteriaceae*. The dominant genera are *Streptomyces*, *Escherichia* and *Bacillus*. Among them, 10 strains with a maximum similarity of less than 98.65% are suspected potential new species. For specific strain information, please refer to Table S1.

Different treatment groups had significant effects on the structure and abundance of microbial communities. The group with norfloxacin and amoxicillin added exhibited the most favorable conditions for microbial growth, especially promoting massive reproduction of the genus *Streptomyces*. As the control group, the CK group had significantly inhibited microbial growth, and the abundance and diversity of the community were the lowest. Each treatment group shaped a unique microbial community composition by altering environmental conditions and other factors, providing basic data for further exploration of the relationship between treatment factors and the response mechanism of microbial communities.

### 3.3. Results of Drug Resistance Testing

**Figure 5:**
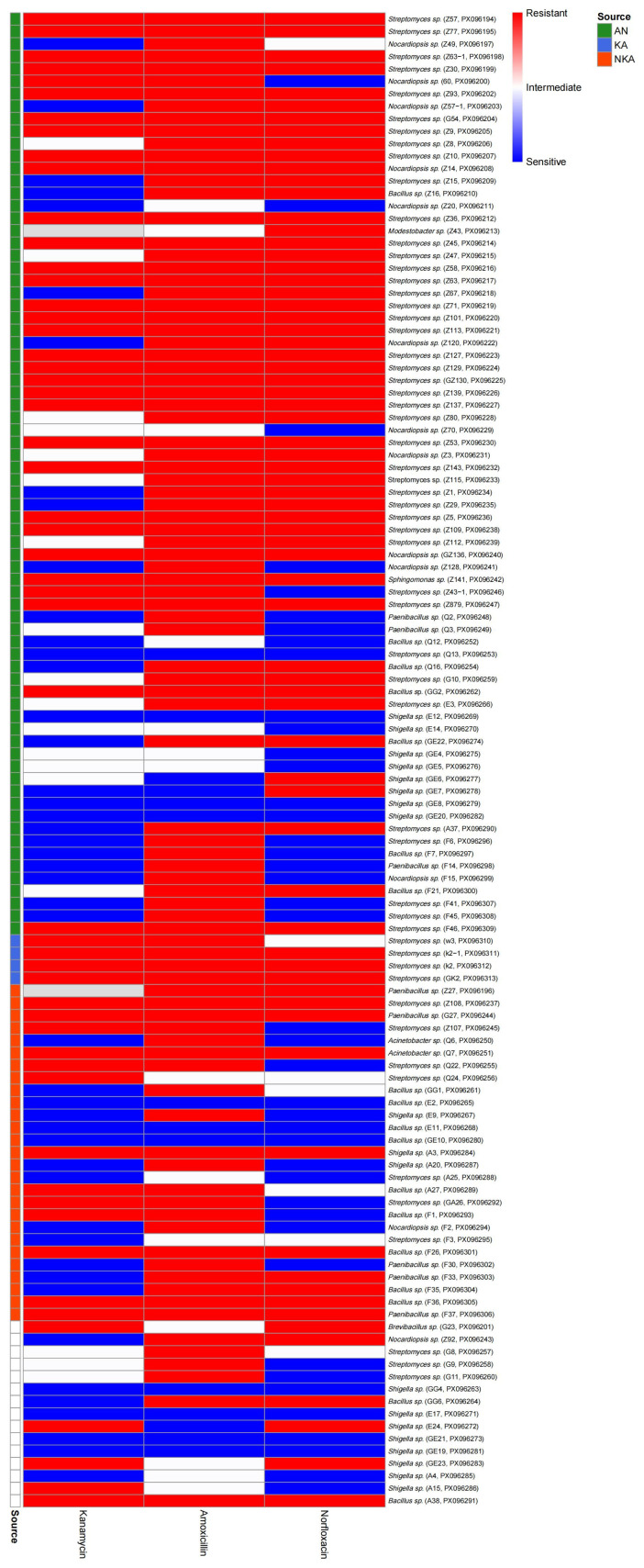
Heatmap of antibiotic resistance profiles grouped by strain source. The heatmap illustrates the antibiotic resistance profiles of 120 bacterial strains isolated from media supplemented with different antibiotic combinations. Each row represents a single strain, with colored sidebars indicating the source group (AN, KA, NKA). The columns correspond to resistance against kanamycin, amoxicillin, and norfloxacin. The color gradient indicates the resistance level: blue for sensitive (S), gray for intermediate (I), and red for resistant (R). Most strains exhibited high levels of resistance to amoxicillin, and multidrug resistance (MDR) was prevalent across groups. This figure highlights the diversity and distribution of resistance patterns among strains from different sources.

A total of 120 bacterial strains were isolated from media containing different combinations of antibiotics. The antibiotic susceptibility test results of the three antibiotics (kanamycin, amoxicillin, and norfloxacin) are shown in Figure 5. The results showed that a total of 118 strains were obtained in the medium containing amoxicillin, among which 91 strains were drug-resistant and 13 strains were mediating, with a drug resistance rate as high as 88.14%. A total of 117 strains were obtained in the medium containing kanamycin, among which 54 were drug-resistant and 17 were moderately drug-resistant, with a drug resistance rate of 60.68%. A total of 119 strains were isolated from the medium containing norfloxacin, among which 70 were drug-resistant and 7 were mediating, with a drug resistance rate of 64.71%.

The heatmap further shows the distribution of resistance spectra and source grouping differences in each strain under the three antibiotics (Figure 5). Most strains showed high resistance to amoxicillin, and some strains were resistant to two or three antibiotics simultaneously, indicating the widespread existence of multidrug resistance (MDR). The distribution of drug resistance of strains from different sources (AN, KA, NKA) under various antibiotics also shows certain heterogeneity. Among them, the resistance spectrum of the AN group is broader, and there are also some sensitive or mediator strains in the KA group and the NKA group. The overall results show that the overall drug resistance level of the isolated bacterial population is relatively high, and the phenomenon of multidrug resistance is significant.

### 3.4. Results of Antibacterial Activity Test

**Figure 6:**
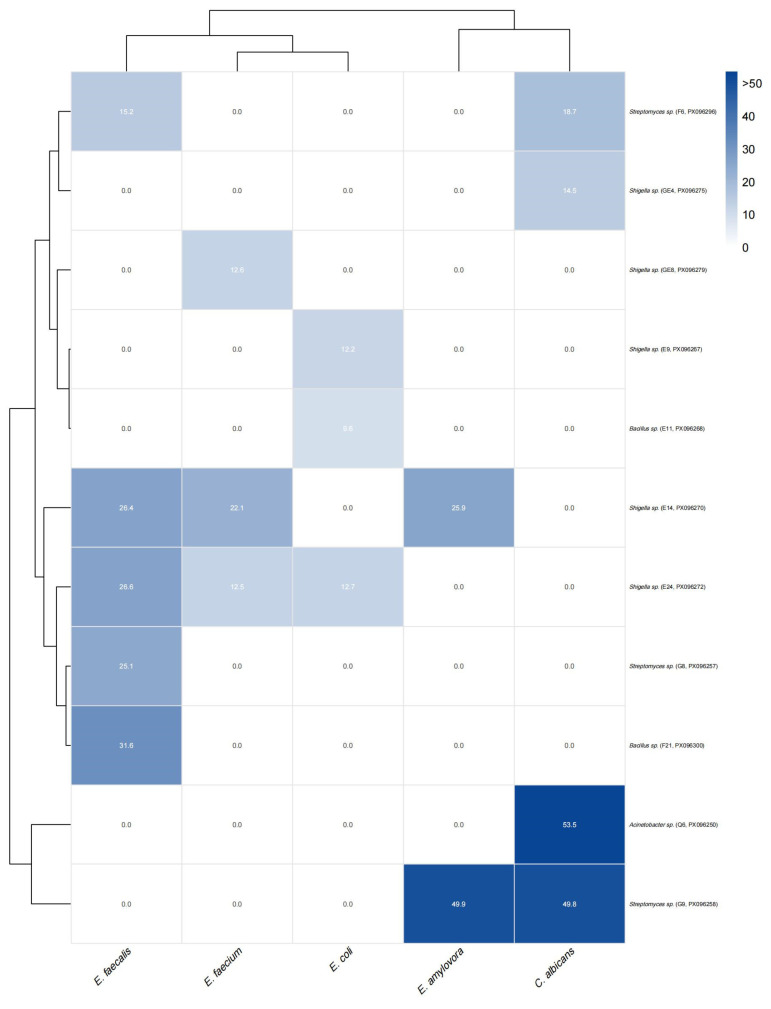
Heatmap of antibacterial activity of bacterial isolates against five pathogens. The heatmap illustrates the inhibition zone diameters (mm) of 12 bacterial isolates (rows) against five target pathogens (columns): *E. faecalis*, *E. faecium*, *E. coli*, *E. amylovora*, and *C. albicans*. Color intensity represents the strength of antibacterial activity, with darker blue indicating a larger inhibition zone and stronger antibacterial effect. White indicates no inhibition (0 mm). Values in each cell denote the mean diameter of the inhibition zone. Both strains and pathogens were clustered based on Euclidean distance and the complete linkage method. Notably, isolates Q6 and G9 showed broad-spectrum and strong antibacterial activity, particularly against *E. amylovora* and *C. albicans*, whereas several other isolates exhibited narrow or no activity.

To evaluate the antagonistic potential of the isolates, 120 microorganisms were screened against five target pathogens using the agar diffusion method (Figure 6). Eleven strains exhibited inhibitory activity, with inhibition zones ranging from 9.57 to 53.51 mm. *E. faecalis* was inhibited by five strains (F6, E14, E24, G8, F21), with F21 showing the strongest effect (31.59 mm). Four strains (F6, GE4, Q6, G9) acted against *C. albicans*, among which Q6 had the largest inhibition zone (53.51 mm). Q6 and G9 were also effective against *E. amylovora* (49.94 mm), while *E. faecium* was inhibited by E14, E24, GE8, and G8 (maximum 22.05 mm). Three strains (E9, E11, E24) acted on *E. coli*, but with weaker inhibition (≤12.74 mm). Notably, E14 and E24 inhibited three pathogens simultaneously, whereas Q6 and G9 displayed strong broad-spectrum activity.

Cluster analysis confirmed these patterns (Figure 6). *E. faecalis* and *E. faecium* clustered together, *C. albicans* and *E. amylovora* formed another group, while *E. coli* separated independently. On the strain side, F6, GE4, G8, F21, E14, and E24 grouped as moderate Gram-positive inhibitors, E9 and E11 clustered due to selective *E. coli* activity, and Q6 and G9 formed a distinct group characterized by broad-spectrum inhibition, with inhibition zones >50 mm. These results highlight functional differentiation among isolates, distinguishing narrow-spectrum from broad-spectrum antibacterial activity.

### 3.5. Correlation Analysis Between Drug Resistance Score and Antibacterial Activity

**Figure 7:**
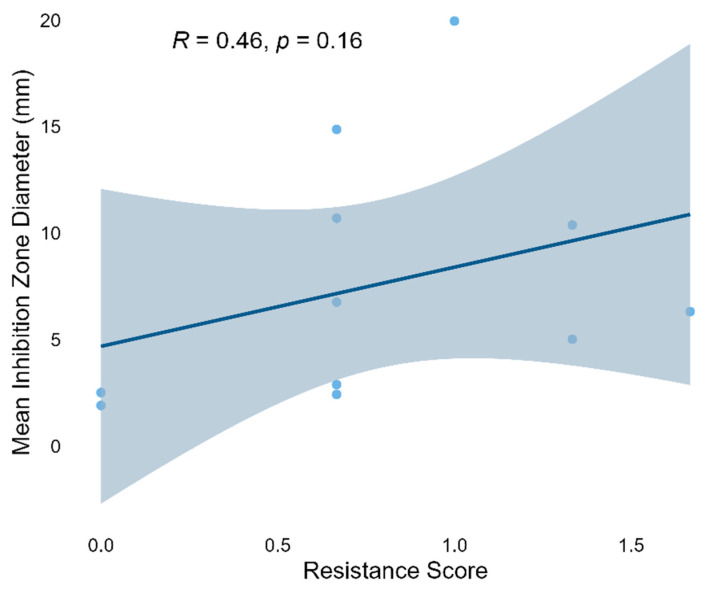
Correlation between resistance score and mean inhibition zone diameter of the isolates. The regression line (blue) with 95% confidence interval (shaded area) is shown (R = 0.46, *p* = 0.16).

To evaluate the relationship between bacterial drug resistance and its antibacterial activity, based on the set weights (R = 2, I = 1, S = 0), the weighted drug resistance score of each strain was successfully calculated for correlation analysis. Figure 7 shows the correlation between the weighted resistance score and the average inhibition zone diameter. Spearman correlation analysis indicates that the resistance score is positively correlated with the average inhibition zone diameter, but not significantly (*ρ* = 0.46, *p* = 0.16). This indicates a weak trend that strains with higher resistance levels tend to show slightly stronger inhibitory effects on the tested pathogens, although the results are not statistically significant. The observed relationship indicates that there may be a decoupling between the resistance and antagonistic abilities of these strains, and further research with larger datasets is needed.

## 4. Discussion

### 4.1. Ecological Link Between Microbial Community Structure and Isolation of Antibacterial Functional Strains

This study revealed the spatial variation in microbial community composition across different regions of Lop Nur and successfully isolated multiple strains with both antibiotic resistance and pathogen-inhibitory activity from nine soil samples. At the community level, *Actinobacteria* and *Firmicutes* were the dominant phyla across all three groups. Consistently, several highly active isolates also belonged to these taxa, notably *Bacillus swezeyi* (e.g., Q6, G9) and *Bacillus australimaris* (e.g., F6, G8, F21), which exhibited broad-spectrum antibacterial activity. These results suggest that these groups not only have colonization advantages in the arid niches of Lop Nur but also possess considerable functional potential.

By integrating amplicon sequencing data with the taxonomic identities of the cultured isolates, we found that certain taxa—such as *Geodermatophilaceae*, *Bacillaceae*, and *Shigella*—were relatively abundant in specific soil samples and also frequently identified among the isolates with antibacterial activity. For instance, *Shigella flexneri* strains showed inhibitory activity against *Candida albicans* and Gram-negative pathogens, implying that antagonistic microbial interactions may naturally occur in desert soils. Previous studies have reported that arid and saline–alkaline environments often drive indigenous microbes to evolve specialized metabolic strategies to cope with environmental stress, such as producing antimicrobial metabolites or forming extracellular polymeric substances as protective matrices [20].

Interestingly, we also observed that the northern region—where overall microbial diversity was relatively low—harbored a greater number of isolates with antibacterial activity. This may indicate that under harsher conditions, certain microbial taxa enhance their metabolic redundancy and resistance functions to stabilize their ecological niches. This observation aligns with the “function–diversity compensation” hypothesis, which posits that in low-diversity ecosystems, some microbes may enhance their functional outputs to compensate for ecological role gaps [21].

Furthermore, our comparative analysis of antibiotic resistance and antibacterial spectrum revealed a potential systemic trade-off: strains with higher resistance scores tended to exhibit smaller average inhibition zones. This suggests that microbes may face a resource allocation dilemma between resistance and antagonism, which is an ecologically and evolutionarily significant phenomenon warranting further investigation [22,23].

In conclusion, the extreme arid soils of Lop Nur not only support distinctive microbial communities but also offer a valuable reservoir for isolating strains with antibiotic resistance and pathogen-inhibitory functions. The observed correlations between microbial community structure and the distribution of functional isolates underscore the key role of indigenous microbiota in environmental adaptation and functional diversification.

### 4.2. Selective Effects of Antibiotic Combinations on Microbial Communities

In this study, we isolated and cultured microorganisms from the Lop Nur soil using various antibiotic regimens (monotherapy and combination therapy). The results showed that antibiotic pressure significantly altered the community structure: the antibiotic-free control group exhibited the highest microbial diversity, with 35 bacterial strains isolated, whereas only 8 strains were recovered under the triple-antibiotic combination (Table 2). This result is consistent with the synergistic antibacterial mechanisms of the antibiotics: norfloxacin targets DNA gyrase in Gram-negative bacteria [22,24], amoxicillin interferes with cell wall synthesis, and kanamycin inhibits protein translation [25]. The combination of all three antibiotics blocks multiple metabolic pathways simultaneously, making it difficult for susceptible strains to survive. Notably, the relative abundance of Gram-negative bacteria increased when norfloxacin was used alone, confirming its strong inhibitory effect on Gram-positive bacteria. In contrast, both Gram-negative and Gram-positive bacteria coexisted in the kanamycin-treated group, likely due to its broad-spectrum action on prokaryotic ribosomes. These observations suggest that antibiotic combinations can serve as targeted selection tools for enriching specific resistant taxa in extreme environments.

In addition, Actinobacteria were enriched under single-antibiotic treatments compared with the control (Table 2), likely due to their intrinsic resistance mechanisms [26] (e.g., efflux pumps, β-lactamases) and competitive advantage after suppression of sensitive taxa. However, their numbers markedly declined under combined-antibiotic treatments, indicating that multidrug pressure exceeded their tolerance capacity.

### 4.3. The Association Between Antibiotic Resistance Traits and Adaptation to Extreme Environments

Microorganisms from Lop Nur exhibited notable resistance to all three antibiotics: the highest resistance rate was observed for amoxicillin (90.29%), followed by norfloxacin (63.16%) and kanamycin (51.02%). Moreover, multidrug resistance was particularly prevalent (Figure 5). This characteristic may be linked to natural selection pressures in extreme environments. The long-term arid and highly saline–alkaline conditions of Lop Nur may mimic antibiotic stress, driving microbes to acquire resistance through genetic mutations (e.g., upregulation of efflux pump genes) or horizontal gene transfer (e.g., acquisition of resistance plasmids) [27,28]. Notably, 9 out of 11 bacterial strains with antibacterial activity also exhibited antibiotic resistance (81.82%), suggesting a potential co-evolutionary link between resistance mechanisms and antimicrobial compound biosynthesis pathways. For instance, certain strains may degrade amoxicillin by producing β-lactamases [29] while simultaneously synthesizing lipopeptide compounds to inhibit pathogens [30]. This “resistance–production” metabolic coupling model offers a novel perspective for the development of antimicrobial agents.

### 4.4. Metabolic Trade-Off Between Multidrug Resistance and Antibacterial Activity

Although a weak positive correlation was observed (Spearman’s *ρ* = 0.46), it did not reach statistical significance (*p* = 0.16). This trend contrasts with conventional views, which generally suggest that acquiring antibiotic resistance often incurs a “fitness cost,” typically manifested as reduced growth rate, diminished metabolic output, or impaired secondary metabolism [23,31]. For example, Andersson and Hughes (2010) noted that in the absence of antibiotics, resistance mutations can impair bacterial physiological functions [31]. Similarly, in *Pseudomonas fluorescens*, resistance-acquiring mutations can suppress the production of antimicrobial secondary metabolites such as phenazines [32].

However, an increasing number of studies suggest that the trade-off between resistance and function is not absolute. Some multidrug-resistant (MDR) strains exhibit enhanced virulence or metabolic capacity alongside their resistance, which may be attributed to compensatory mutations or the co-selection of resistance and metabolic genes [23,31]. In soil bacteria such as *Bacillus subtilis*, β-lactam resistance is closely associated with the high expression of antimicrobial lipopeptides [33]. Moreover, microorganisms in natural environments—particularly those from soil and the plant rhizosphere—are often simultaneously exposed to antibiotic pressure and intense competition for survival, making them more likely to evolve strategies that integrate both resistance and antagonistic capabilities [34].

Although a positive correlation was observed, its lack of statistical significance suggests that the phenomenon may be strain-specific or environmentally dependent. Expanding the strain sample size and integrating metabolomic data with functional genomic annotation will be valuable for elucidating the systematic relationship between antibiotic resistance and antibacterial activity.

### 4.5. Ecological Significance and Applied Potential of Antibacterial Activity

In this study, a total of 11 bacterial strains were identified with antibacterial activity against common pathogens, including *Staphylococcus aureus*, *Escherichia coli*, and *Candida albicans*, with the largest inhibition zone reaching 53.51 mm—demonstrating substantial antimicrobial potential. Notably, strains E24 and E14 were capable of inhibiting all three pathogens simultaneously, suggesting that their metabolites possess broad-spectrum activity, potentially involving multi-target or non-specific antimicrobial factors. Resistance profiling further revealed that strains exhibiting multidrug resistance also showed enhanced antibacterial activity, particularly those isolated from the antibiotic combination treatment group, which generally displayed broader inhibitory spectra. This observation indicates that antibiotic pressure may not only act as a selective force enriching resistant populations but also facilitate the co-evolution of “high-resistance–high-activity” microbes, offering new insights into the discovery of natural antimicrobial agents.

Ecologically, such microorganisms often gain a competitive advantage in resource-limited extreme environments by synthesizing antimicrobial compounds. These metabolites serve as key biotic antagonistic factors that suppress potential competitors or pathogens [35]. For example, *Actinobacteria* and *Bacillus* species are prolific producers of peptide- and polyketide-based natural antibiotics, which have been shown to play critical ecological roles in natural soils and the plant rhizosphere [36]. In agricultural applications, such antagonistic strains can be developed into biocontrol agents to replace or reduce the use of chemical pesticides, thereby enhancing crop health and minimizing environmental pollution. In the medical field, microbial products derived from extreme environments hold promise as novel drug candidates against multidrug-resistant pathogens, owing to their structural novelty and diverse modes of action.

Therefore, the strains isolated in this study that possess both antibiotic resistance and antimicrobial production capabilities are not only ecologically significant for adaptation but also exhibit substantial practical application potential. Future work should integrate metabolomics and structural analysis techniques to comprehensively explore the diversity and mechanisms of their bioactive metabolites, thereby advancing the translational application of extremophile microbial resources into novel antimicrobial agents.

### 4.6. Co-Occurrence of Functional Genes and Mechanisms of Co-Evolution

In extreme environments, microorganisms are simultaneously exposed to salinity, radiation, and nutrient scarcity, and their metabolic networks often respond through the coordinated activity of functional gene modules rather than isolated pathways. Previous studies have shown that resistance genes—such as those encoding multidrug efflux pumps (e.g., the acrAB-tolC system)—are frequently co-located with antimicrobial biosynthetic genes, including polyketide synthases (PKSs) and nonribosomal peptide synthetases (NRPSs), or co-expressed under shared regulatory control [37,38]. This arrangement reflects the gradual evolution of “metabolic synergy units,” enabling microbes to withstand complex stresses.

In light of the findings from this study, under triple-antibiotic stress from norfloxacin, kanamycin, and amoxicillin, certain multidrug-resistant (MDR) strains—such as *Bacillus swezeyi* Q6 and G9—not only exhibited high levels of resistance but also demonstrated broad-spectrum antibacterial activity, suggesting the potential for coordinated expression of both resistance and antimicrobial production traits. This phenomenon may be explained by the following mechanism: activation of efflux pumps may not only expel harmful antibiotic molecules but also facilitate the efficient secretion of endogenous antimicrobial metabolites, thereby enhancing ecological competitiveness [39]. On the other hand, the promoter regions of relevant genes may share common stress-responsive regulatory elements—such as σ factor recognition sites or MarR family binding sequences—that enable their simultaneous activation under salt–alkaline, antibiotic, or other stress conditions, thereby facilitating coordinated expression of associated metabolic pathways [40].

Moreover, in certain *Streptomyces* and *Bacillus* species, the co-occurrence of β-lactam resistance and the ability to synthesize antimicrobial lipopeptides has been observed. This suggests that these microbial groups may employ a co-evolutionary strategy based on “gene cluster co-expression + shared regulatory elements,” integrating resistance and antimicrobial activity within a unified metabolic framework to enhance both environmental adaptability and ecological competitiveness [41]. This modular and co-expressed metabolic model not only offers new insights into the adaptive mechanisms of extremophilic microorganisms but also provides a theoretical foundation for the discovery of natural product resources.

Further validation of the proposed mechanisms will require follow-up genomic sequencing and transcriptomic expression profiling. For example, comparative analysis of the genomic architecture and gene expression patterns between the 11 strains exhibiting antibacterial activity and strains without antibacterial activity identified in this study and strains without antibacterial activity may help uncover potential co-regulated functional modules and key metabolic pathways [42,43]. This research direction not only contributes to elucidating the evolutionary coupling between antibiotic resistance and secondary metabolite production but also lays the groundwork for understanding the universality and adaptive significance of functional gene co-occurrence in extreme environments.

### 4.7. Limitations of the Study and Future Perspectives

This study has two main limitations. First, the use of traditional cultivation methods likely excluded approximately 99% of unculturable microorganisms, underscoring the need to integrate metagenomic approaches to comprehensively resolve community diversity. Second, the current analysis is limited to the phenotypic level, without in-depth investigation into the molecular mechanisms of resistance genes (e.g., *bla*, *mcr*) and antimicrobial biosynthetic genes (e.g., *NRPS*, *PKS*). It should be noted that the present study relied on agar diffusion assays for preliminary screening of antibacterial activity. Although inhibition zone diameters provide useful comparative data, they do not yield standardized quantitative values. Determination of minimum inhibitory concentrations (MICs) through broth dilution or microdilution assays was beyond the scope of this study due to resource limitations, but will be performed in future work to further validate and extend the antibacterial potential of the isolates. Moreover, the long-term overuse of antibiotics in extreme environments such as Lop Nur may reduce microbial biodiversity, enrich resistant taxa, and compromise ecosystem resilience, an issue that requires systematic long-term monitoring and multi-omics approaches in future studies.

Future research should expand the sampling scope (e.g., deeper soil layers, diverse geomorphological units) and apply multi-omics techniques—including transcriptomics and metabolomics—to systematically uncover the co-evolutionary mechanisms underlying the interplay between antibiotic resistance and antibacterial activity. Additionally, evaluating the colonization potential and ecological risks of resistant strains in natural environments will provide theoretical support for their safe application. As one of the Earth’s regions most analogous to the Martian environment, the microbial resistance mechanisms shaped by long-term natural selection in Lop Nur may preserve ancestral evolutionary pathways, offering key insights into the “non-clinical origins” of antibiotic resistance. However, whether microbial resistance in such pristine, antibiotic-unpolluted extreme environments arises from intrinsic resistance (e.g., ancient resistance genes) or from acquired resistance induced by natural chemical stresses (e.g., high salinity, heavy metals) remains to be systematically investigated.

## 5. Conclusions

This study systematically evaluated the antibiotic resistance and antibacterial activity of soil microorganisms from the extremely arid and saline–alkaline environment of Lop Nur, and explored the potential functional coupling between these traits. A total of 120 bacterial strains were isolated, spanning six phyla and nine genera, including 10 potential novel species, highlighting the region’s rich microbial diversity. The combined use of three antibiotics significantly altered microbial community composition, exhibiting synergistic inhibitory effects and effectively enriching specific resistant taxa. Resistance profiling revealed the highest resistance rate against amoxicillin, with widespread multidrug resistance, indicating strong microbial adaptability to compound stress. Certain strains, such as *Bacillus swezeyi* Q6 and *B. australimaris* F21, displayed both multidrug resistance and broad-spectrum antimicrobial activity, suggesting a possible co-regulation of resistance and antimicrobial production (“resistance–biosynthesis” coupling). Future studies using genomic and transcriptomic approaches to compare strains with and without antibacterial activity could help identify co-expressed gene modules and validate underlying metabolic synergy. This work provides a theoretical foundation for the discovery of novel antimicrobial agents and advances our understanding of functional evolution in extremophilic microorganisms.

## Figures and Tables

**Table 1:** Sample collection information.

No.	A	B	C
Direction	South	Central	West
Sample types	Sandy	Sandy	Sandy
Longitude	89°27′22″ N	90°57′43″ N	91°51′39″ N
Latitude	39°16′18″ E	40°36′04″ E	41°39′00″ E
Altitude (m)	1067	730	1114

**Table 2:** The impact of adding antibiotics on isolation and culture.

Antibiotics	Colonies Number (CFU)		
	Actinobacteria	Bacteria	Fungi
No Antibiotics Added	8	35	5
Norfloxacin	14	5	1
Kanamycin	16	4	0
Amoxicillin	13	9	1
Norfloxacin + Kanamycin	4	2	0
Norfloxacin + Amoxicillin	5	6	0
Kanamycin + Amoxicillin	1	3	0
Norfloxacin + Kanamycin + Amoxicillin	2	2	0

## Data Availability

The datasets generated for this study can be found in GenBank under the accession numbers PX096194-PX096313.

## Supplementary Materials

The following supporting information can be downloaded at https://www.mdpi.com/article/10.3390/microorganisms13092076/s1: Table S1: Information on strains isolated under antibiotic selection.
